# Enhanced Photocatalytic CO2 Reduction Performance by External Electric Field‐Driven Charge Separation on Interdigitated Micro‐Spacing Structure

**DOI:** 10.1002/advs.202523076

**Published:** 2026-01-11

**Authors:** Xidan Tang, Zhenyu Yu, Jie Liang, Xiaoguang Wang, Qin Shi, Honghui Pan, Xixiang Liu, Pengyi Tang

**Affiliations:** ^1^ School of Materials and Environment, Guangxi Key Laboratory of Advanced Structural Materials and Carbon Neutralization, Guangxi Engineering Research Center for Advanced Materials and Intelligent Manufacturing Guangxi Minzu University Nanning China; ^2^ National Key Laboratory of Materials for Integrated Circuits and 2020 X‐Lab, Shanghai Institute of Microsystem and Information Technology Chinese Academy of Sciences Shanghai China; ^3^ Hubei Key Laboratory of Environmental and Health Effects of Persistent Toxic Substances, Hubei Key Laboratory of Industrial Fume and Dust Pollution Control, School of Environment and Health Jianghan University Wuhan China

**Keywords:** charge separation, external electric field, interdigitated micro‐spacing, photocatalytic CO_2_ reduction

## Abstract

Traditional photoelectrocatalysis (PEC) separates photogenerated charge carriers via an external bias. However, its complex configuration and high‐resistance liquid electrolytes restrict its application largely to liquid‐phase systems. Taking advantage of the remarkable enhancement of the electric field gradient enabled by the interdigitated electrodes (IDEs) with a spacing of 100 µm, an external electric field‐enhanced photocatalytic CO_2_ reduction system was developed. A relatively high electric field strength can be generated under the application of a small external voltage. Given that the electric field strength is inversely proportional to the electrode spacing, when a 1.0 V is applied to electrodes with a 100 µm spacing, the electric field strength can reach the order of 104 V m^−1^. By directly applying a modest external voltage (0.5–1.5 V) to hydrogenated TiO_2_ photocatalysts, an externally induced electric field drives the directional migration of charge carriers, enhancing the separation and transport of photogenerated carriers. At 1.5 V applied voltage, the hydrogenated TK_450_ catalyst achieves CH_4_ and C_2_H_6_ production rates of 31.1 and 4.9 µmolg^−1^ h^−1^, represents a four‐fold enhancement compared to the pristine TiO_2_ baseline. Consequently, the application of this electric field‐enhanced photocatalytic system to gas‐phase reaction for addressing energy and environmental challenges.

## Graphical abstract

An external electric field‐enhanced photocatalytic system is developed using interdigitated electrodes with 100 µm spacing to overcome the liquid‐phase limitations of traditional PEC. The induced high‐intensity electric field effectively drives the directional migration of charge carriers under low voltage. This strategy significantly suppresses charge recombination and achieves a four‐fold enhancement in CH_4_ production over hydrogenated TiO_2_, offering a new approach for efficient gas‐phase CO_2_ reduction.

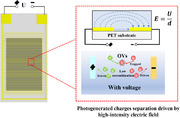

## 1 Introduction

The accelerated progression of global industrialization has driven extensive fossil fuel consumption, resulting in a substantial surge in anthropogenic CO_2_ emissions [1]. Current global CO_2_ emissions have reached 37.4 gigatons annually [2], Consequently, the resulting global warming crisis has emerged as a major focus of international scientific and policy discourse [3]. To achieve the critical objective of carbon neutrality, semiconductor‐mediated photocatalytic CO_2_ reduction represents a promising technological pathway for synchronously addressing greenhouse gas mitigation and sustainable fuel production [4]. This approach utilizes solar energy to drive the thermodynamically uphill conversion of CO_2_ into value‐added hydrocarbons [5], thereby offering a synergistic solution to the dual crises of energy security and environmental sustainability.

The efficiency of the photocatalytic CO_2_ reduction reaction is significantly constrained by the dynamics of photogenerated carrier complexes. It has been demonstrated that the lifetimes of electron‐hole pairs generated by photoexcitation are typically in the range of picoseconds to microseconds, and their effective diffusion distances are predominantly constrained to the nanometer scale [6, 7]. In charge carrier transport‐dominated photocatalytic reactions, the majority of photogenerated electrons and holes, influenced by mutual Coulombic interactions, undergo rapid radiative recombination in the bulk phase. Specifically, the recombination rate of photogenerated electron–hole pairs in the bulk phase significantly exceeds their migration rate to the surface for reaction participation. This directly diminishes the concentration of charge carriers available for surface catalytic reactions, resulting in quantum efficiency remaining far below the theoretical limit [8]. This is particularly evident in the deep reduction reaction of CO_2_ with multiple electron transfers, where the sustained supply ability of carriers becomes a pivotal factor in determining the product selectivity. In photocatalytic CO_2_ reduction reactions, the separation efficiency of photogenerated carriers is further optimized under the application of a moderate external voltage [9]. The imposed electric field drives photogenerated electrons and holes to migrate in opposite directions, effectively suppressing their recombination [10, 11]. This electro‐assisted photocatalytic system enhances CO_2_ reduction performance without altering the intrinsic properties of the catalyst [12], demonstrating its potential for scalable solar fuel production.

PECS technology enables directional transport of photogenerated charge carriers and spatial decoupling of redox reactions via synergistic light‐electric field engineering [13]. In conventional aqueous‐phase PEC systems, the semiconductor photoanode and metallic counter electrode are immersed in liquid electrolytes to establish ionic conduction pathways for interfacial charge transfer. However, some intrinsic limitations constrain its practical implementation: 1) The requisite liquid electrolyte imposes multiphase mass transfer barriers of gas‐to‐liquid dissolution, and complex reactor configurations, collectively degrading interfacial reaction kinetics. 2) The substantial charge transfer overpotential at the solid‐liquid interface necessitates additional bias voltages, which concomitantly escalates energy input and triggers parasitic reactions (e.g., hydrogen evolution reaction [HER], oxygen evolution reaction [OER]). 3) Prolonged operation induces synergistic degradation mechanisms through anodic corrosion by aggressive electrolytes and photocorrosion under illumination [14, 15].

Gas‐phase photoelectrocatalysis (GPEC) demonstrates transformative potential through its ability to overcome critical limitations of conventional systems. Recent advancements adapted solid‐phase designs using platinum counter‐electrodes or solid‐state electrolytes [16, 17]. However, a portion of the voltage dissipation at semiconductor‐electrolyte interfaces persisted [18, 19], compromising scalability. Previous work demonstrated that a modest external voltage (0.5–1.5 V) applied to monolithic semiconductor photocatalysts induces an electric field driving directional carrier migration. This field‐enhanced charge transport increases carrier density at reactive sites, resulting in a 3.5‐fold photocatalytic performance enhancement [20]. This system diverges from conventional photoelectrocatalytic architectures by eliminating the need for a counter electrode and electrolyte. Moreover, the voltage is applied directly to the photocatalyst, achieving substantial performance enhancement under a modest external bias. Preliminary investigations have revealed that the PEC performance is fundamentally governed by three interdependent parameters in the system: charge carrier concentration (determining photogenerated electron–hole pair density under illumination), carrier mobility (controlling intrinsic transport properties), and applied electric field strength (regulating directional migration distance). While the former two are material‐inherent characteristics, the electric field can be precisely modulated via bias voltage and electrode spacing optimization [21]. In light of these principles, we engineered an interdigitated electrode architecture with microscale spacing (1–100 µm) that harnesses edge‐field effects to amplify local electric fields. Theoretically, this design can achieve remarkable field intensities (e.g., 10^4^ V m^−1^ at 1.0 V bias across 100 µm gaps) under ultralow voltage conditions, significantly enhancing CO_2_ photoreduction efficiency [22]. The compact electrode configuration simultaneously maximizes active surface area and field gradient density, overcoming traditional trade‐offs between charge transport and mass transfer in gas‐phase systems. However, the correlation between field‐modulated carrier dynamics (mobility/lifetime), localized field strength distribution, and resultant products selectivity remains ambiguous.

Herein, this study introduces a novel electric field‐enhanced photocatalytic CO_2_ reduction system without electrolyte and counter electrode. The reduction effect of sodium borohydride (NaBH_4_) introduces oxygen vacancies (OVs) and Ti^3+^ states into TiO_2_, which alter the electronic structure of TiO_2_, thereby enhancing its carrier concentration and mobility. The hydrogenated TiO_2_ photocatalyst was deposited on interdigitated electrodes (IDEs), and the application of a voltage to the electrodes significantly enhances the efficiency of gas‐solid heterogeneous photocatalytic CO_2_ reduction reactions. Applying a voltage to the IDEs to create a high‐intensity electric field significantly enhanced the efficiency of gas‐solid heterogeneous photocatalytic CO_2_ reduction. Building upon this configuration, transient photocurrent measurements and photoelectrochemical characterization were combined with steady‐state photoluminescence (PL) and time‐resolved photoluminescence (TRPL) to investigate distinct aspects of photoinduced charge dynamics. External electric field enables effective spatial charge separation and suppresses defect‐mediated recombination, thereby substantially improving the photocatalytic activity of the photocatalyst. The electric field‐enhanced strategy resolves this contradiction by optimizing electron transfer kinetics within the photocatalytic system. Elucidating the influence of external electric field on charge transfer dynamics and mechanisms in semiconductors holds critical significance. These findings provide new opportunities for constructing efficient photocatalytic systems with other doped catalysts and facilitate broader applications.

## 2 Results and Discussion

### 2.1 Characterizations of Photocatalysts

High‐resolution transmission electron microscopy (HRTEM) images clearly revealed lattice fringes with an interplanar spacing of 0.35 nm (Figure S2a) [24, 25], which was in excellent agreement with the theoretical value for the (101) plane of anatase TiO_2_. This observation confirmed the presence of the anatase phase and the high crystallinity of the samples. However, localized lattice disorder and missing lattice planes were evident in the HRTEM images of the TK_400_ (Figure S2b) and TK_450_ (Figure S2c) samples. These structural defects are likely attributed to oxygen vacancies and Ti^3+^ species introduced during the thermal reduction process, which not only alter the local electronic structure of TiO_2_ but may also significantly influence its photocatalytic performance.

**FIGURE 1:**
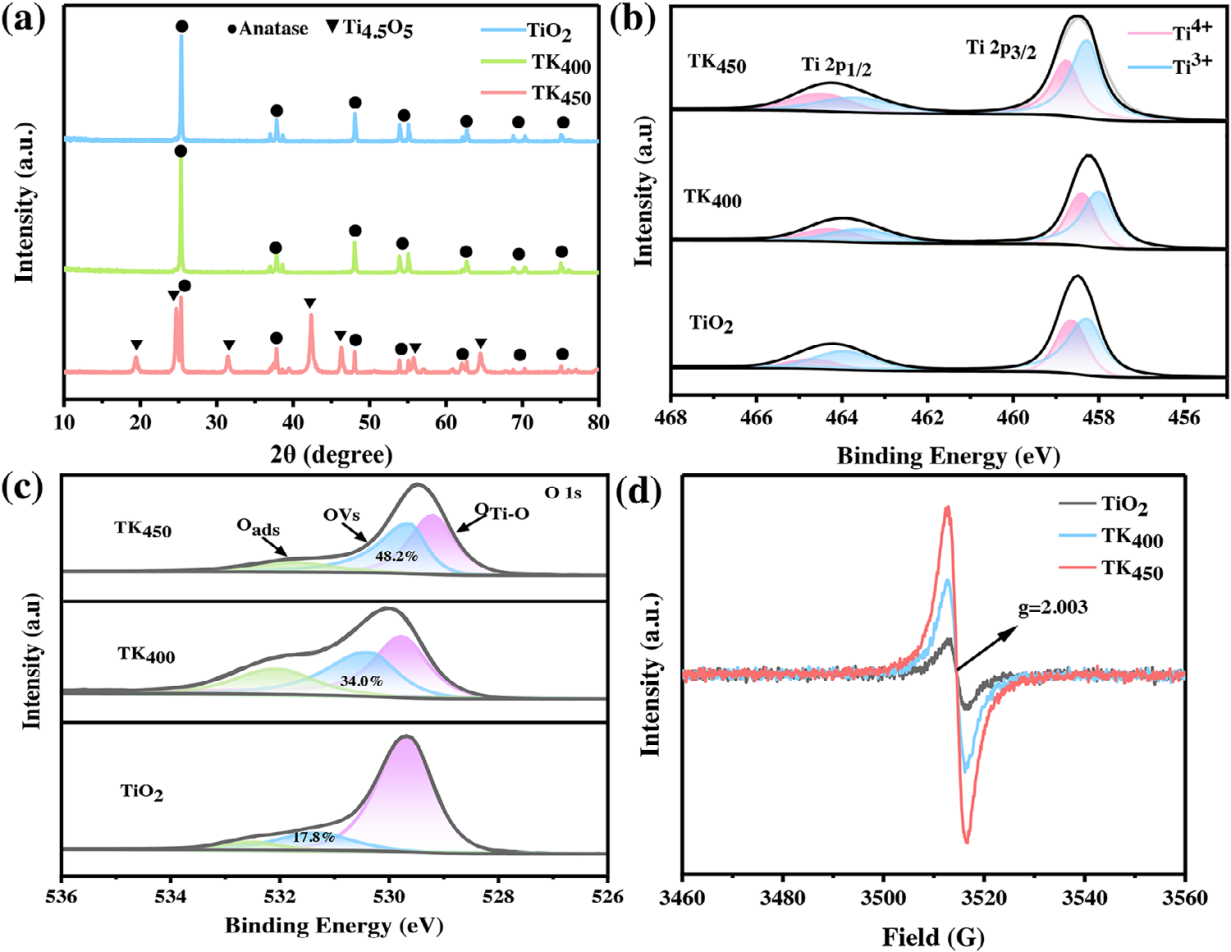
a) XRD patterns of the fabricated catalysts; b) High resolution XPS Ti 2p spectra of catalysts; c) O 1s XPS spectra of catalysts; d) EPR spectra.

To systematically investigate the influence of calcination temperature on the phase composition of TiO_2_, X‐ray diffraction (XRD) characterization was performed under controlled pyrolysis conditions. As illustrated in Figure 1a, the XRD patterns of all TiO_2_ samples exhibited distinct diffraction peaks at 25.3°, 37.9°, 48.1°, 54.0°, 55.1°, 62.8°, 68.9°, and 70.3°, which are assigned to the (101), (004), (200), (105), (211), (204), (116), and (220) crystallographic planes of anatase TiO_2_ (PDF#01‐086‐1157) [26], respectively. Notably, the TK_450_ sample displays additional peaks corresponding to the Ti_4.5_O_5_ phase (PDF#01‐071‐6414), a non‐stoichiometric titanium suboxide. This observation confirmed the partial reduction of Ti^4+^ to Ti^3+^ during pyrolysis under inert atmosphere [11], attributable to oxygen vacancy formation and lattice restructuring.

The surface chemical states and defect structures of the samples were systematically characterized by high‐resolution X‐ray photoelectron spectroscopy (XPS). The high‐resolution XPS spectra of Ti 2p (Figure 1b) demonstrates that the 2p_3/2_ and 2p_1/2_ characteristic peaks of TiO_2_ species are located at 459.2 and 465.1 eV [27], respectively. In contrast, the Ti^3+^ species exhibit peaks at 458.0 eV (2p_3/2_) and 463.9 eV (2p_1/2_) [28], displaying a negative binding energy shift of ca. 1.2 eV relative to the Ti^4+^ peaks. This indicates that the hydrogenation treatment induced a shift of Ti^4+^ to the Ti^3+^ reduction reaction. The relative contents of Ti^4+^ and Ti^3+^ were determined from the peak areas by further peak fitting resolution of the characteristic peaks of Ti^4+^ and Ti^3+^ [29]. As demonstrated in Table S1, the relative proportions of Ti^4+^ and Ti^3+^ in TiO_2_ were 42.0% and 58.0%, respectively. It was observed that the content of Ti^3+^ gradually increased with the increase of pyrolysis temperature, and the maximum relative proportion of Ti^3+^ was elevated up to 80.0% in TK_450_, suggesting that the release of NaBH_4_ by thermally activated NaBH_4_ at 450°C temperature reactive hydrogen to promote Ti─O bond breaking and oxygen vacancy formation [30]. Moreover, the O 1s XPS spectra of samples prepared with TiO_2_ (Figure 1c), at 400 and 450°C. The characteristic peaks located at 529.0, 530.0, and 532.0 eV correspond to Ti─O bonds (O_Ti‐O_), oxygen vacancies (OVs), and adsorbed oxygen (Oads), respectively. A clear trend can be observed: with the increase of temperature, the concentration of oxygen vacancies gradually increases, from 17.8% in the original TiO_2_ to 48.2% in the TK_450_ sample, which intuitively demonstrates the promoting effect of pyrolysis temperature on the formation of oxygen vacancies. Electron paramagnetic resonance (EPR) test results are presented in Figure 1d. The spectrum exhibits a characteristic single‐electron paramagnetic signal at g = 2.003, which is attributed to OVs. Compared to TiO_2_, TK_450_ displays a stronger and more symmetrical resonance peak at a g‑factor of 2.003. This result is consistent with the conclusions derived from the XPS analysis.

### 2.2 Photoelectrochemical Properties

**FIGURE 2:**
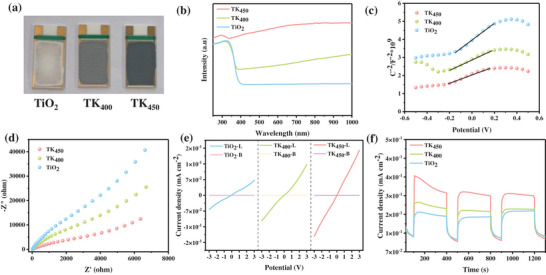
Photoelectrochemical property of as prepared electrodes: a) Catalyst‐coated interdigitated electrodes; b) UV–vis DRS; c) The Mott–Schottky curve; d) EIS of the fabricated catalysts; e) *I–V* curves under dark and illuminated conditions; f) Photocurrent curve at 1.5 V.

After hydrogenation treatment, the sample color changed from white to pale blue, and further deepened to dark blue with increasing hydrogenation temperature (Figure 2a) [23]. The investigation into the light absorption properties was conducted by UV–vis–NIR DRS. As illustrated in Figure 2b, it was demonstrated that the doping of TiO_2_ with abundant Ti^3+^ species enhanced the full‐spectrum absorption, and that the hydrogenated TiO_2_ exhibited a significant photoresponsivity intensity in the vis–NIR region [23]. Furthermore, the TK_450_ sample exhibited the highest absorption intensity in the spectral range of 300–1000 nm, which was attributed to the Ti^3+^‐induced synergistic effect of mid‐gap state and OVs associated charge transfer.

Through systematic optoelectronic characterization, this study elucidates the regulatory mechanism of hydrogenation treatment on the electronic structure of TiO_2_ and its impact on photogenerated carrier transport characteristics. Hall effect measurements reveal that hydrogenation treatment significantly enhances the carrier concentration (N_D_) from 2.2 × 10^19^ cm^−3^ in pristine TiO_2_ to 4.1 × 10^19^ cm^−3^ (Figure 2c) [8]. This phenomenon exhibits strict quantitative consistency with the Mott–Schottky‐derived carrier concentration distribution TiO_2_: 2.7 × 10^19^ cm^−3^; TK_400_: 4.0 × 10^19^ cm^−3^; TK_450_: 4.7 × 10^19^ cm^−3^, confirming that hydrogenation induces synergistic effects between OVs and Ti^3+^ defects. These defects form shallow donor levels, effectively reducing the energy gap between the Fermi level and conduction band minimum, thereby enhancing carrier density. Further analysis of carrier mobility (µ) reveals that the TK_450_ sample achieves µ = 10.5 cm^2^ V^−1^ s^−1^, representing a 2‐fold enhancement compared to pristine TiO_2_ (4.42 cm^2^ V^−1^ s^−1^). This dual improvement in both carrier concentration and mobility facilitates charge separation while suppressing recombination [31], thereby elevating reaction efficiency. The calculation of the conductivity, as outlined in Equation (2) [32], indicates an enhancement of 4.5‐fold in the σ value of TK_450_, as compared to the conductivity of 15.4 S cm^−1^ in pristine TiO_2_. The present study systematically evaluated the charge separation and transfer performance of different catalysts under light conditions by means of Electrochemical Impedance Spectroscopy (EIS) testing [33]. As demonstrated in Figure 2d, the TK_450_ sample exhibited the smallest arc radius in the Nyquist plot, a result that was significantly better than that of the other catalysts (e.g., TiO_2_ and TK_400_). This finding indicates that TK_450_ exhibits superior charge separation and transfer performance. This suggests that the hydrogenation treatment optimizes the charge transport capability while maintaining the intrinsic properties of the material. It is noteworthy that the enhancement in conductivity directly correlates with the spatial separation efficiency of the photogenerated carriers [34]. The high carrier concentration reduces the semiconductor bulk phase resistance, thereby enabling the external voltage to drive the directional carrier migration more efficiently. The *I–V* curves of were plotted by linearly increasing the bias potential to 3.0 V between the photoelectrodes and the counter electrode (Figure 2e). It is evident from the *I–V* curves that a weak current response is observed under dark conditions. However, the addition of light resulted in a higher photocurrent response from the photoelectrodes of T_L_, TK_400‐L_, TK_450‐L_. The experimental findings demonstrated that the photocurrent of TK_450_ was 1.65 × 10^−1^ mA cm^−2^ at 3.0 V higher than that of the undoped TiO_2_ samples of 5.40 × 10^−2^ mA cm^−2^, indicating that the formation of TK_450_ material not only enhanced the photoresponse but also increased the surface reaction activity. The transient photocurrent response plots of TiO_2_, TK_400_, and TK_450_ under the irradiation was displayed in Figure 2f. The TK_450_ sample exhibited a photocurrent density of 0.36 mA cm^−2^, demonstrating a 100.0% enhancement compared to TiO_2_ (0.18 mA cm^−2^) after hydrogenation treatment. This improvement indicated prolonged photoexcited charge carrier lifetimes and enhanced photocatalytic activity of the material [35]. Under repeated on‐off intermittent illumination cycles, the photocurrent decay remains minimal, confirming the robust stability of the photocurrent generation in TK_450_.

(1) $$\sigma =N_{D}e\mu$$

### 2.3 Electric Field‐Enhanced Photocatalytic CO_2_ Reduction

**FIGURE 3:**
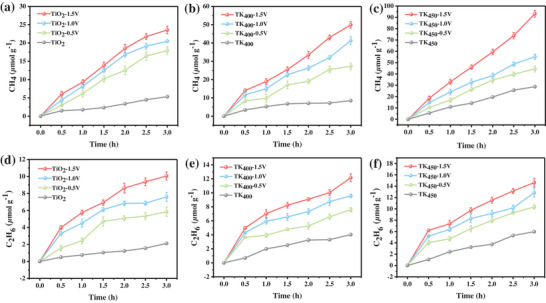
Cumulative evolution of a,b,c) CH_4_ and d,e,f) C_2_H_6_.

In order to verify the feasibility of electric field‐enhanced photocatalytic CO_2_ reduction, the external voltage (0–1.5 V) was applied to the interdigitated electrode substrate coated with catalyst material. The effect of the applied voltage magnitude on the catalytic performance was then tested under UV illumination conditions. As demonstrated in Figure 3a,d, the yields of CH_4_ and C_2_H_6_ in pure TiO_2_ during the reaction were 5.3 and 2.1 µmol g^−1^ in the absence of applied voltage, and the yields of CH_4_ and C_2_H_6_ increased to 23.6 and 10.1 µmol g^−1^ with the application of an external voltage ranging from 0 V to 1.5 V, exhibiting a substantial enhancement. As demonstrated in Figure 3b,c,e,f, the TK_400_ and TK_450_ catalysts exhibited significantly divergent CO_2_ reduction performance under external voltage assisted conditions. Specifically, TK_400_ catalyzed the conversion of CO_2_ to CH_4_ and C_2_H_6_ with maximum yields of 49.9 and 12.2 µmol g^−1^, respectively. In contrast, TK_450_ subjected to a higher‐temperature hydrogenation treatment achieved higher product yields, corresponding to CH_4_ and C_2_H_6_ yields of 93.6 and 14.2 µmol g^−1^, respectively.

**FIGURE 4:**
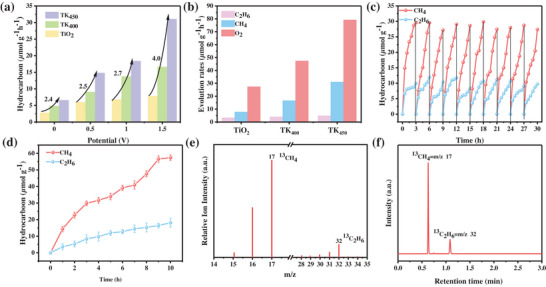
a) Rate of hydrocarbons production for each catalyst applied by different external voltages; b) Product evolution rates under 1.5 V applied voltage; c,d) Photocatalytic stability test of the TK_450_ catalyst with 1.5 V external voltage; e) MS signals of the ^13^CO_2_ isotope labelling experiment.

The electric field‐enhanced photocatalytic system achieved a remarkable hydrocarbons production rate from CO_2_ conversion [10]. As illustrated in Figure 4a, the hydrocarbon (CH_4_/C_2_H_6_) yields of pristine TiO_2_, hydrogenated TK_400_ and TK_450_ catalysts under electrically‐assisted photocatalytic CO_2_ reduction conditions are shown as a function of voltage. It is evident that all catalysts demonstrate heightened activity, attributable to the electric field‐driven charge separation phenomenon [36], when the applied potential is elevated from 0 to 1.5 V. The catalysts also demonstrate enhanced reactivity in comparison to the original TiO_2_. Pristine TiO_2_ exhibits a modest enhancement, with yields increasing from 2.8 µmol g^−1^ h^−1^ (0 V) to 7.9 µmol g^−1^ h^−1^ (1.5 V). In contrast, the performance of the hydrogenated samples was much higher: under the same conditions, TK_400_ increased from 4.8 µmol g^−1^ h^−1^ (0 V) to 16.6 µmol g^−1^ h^−1^ (1.5 V), Notably, the AQY for TK_450_ correspondingly increased from 0.1% to 0.5% as the hydrocarbon production rate rose from 6.6 µmol g^−1^ h^−1^ (at 0 V) to 31.1 µmol g^−1^ h^−1^ (at 1.5 V). It is particularly noteworthy that the hydrocarbon generation rate from CO_2_ conversion achieved by the TK_450_ sample at 1.5 V represented a four‐fold enhancement compared to that of the TiO_2_ reference. The valence band‐driven water oxidation half‐reaction on the photocatalyst serves as a critical process during photocatalytic CO_2_ reduction, not only supplying protons for CO_2_ hydrogenation but also enabling O_2_ evolution. Quantitative verification of the quasi‐stoichiometric electron transfer balance between reduction and oxidation products reveals that the TK_450_ catalyst achieves an O_2_ evolution rate of 79.1 µmol g^−1^ h^−1^ under 1.5 V applied voltage (Figure 4b).

This discrepancy in performance can be attributed to the modulation of the catalyst's structural properties by the hydrogenation treatment temperature. Conversely, the enhanced conductivity and carrier concentration of TK_450_ and TK_400_ following self‐doping treatment leads to increased photogenerated carrier migration to the catalyst surface, where they react with CO_2_ and H_2_O molecules under constant light conditions, as observed for TiO_2_. Consequently, TK_450_ demonstrates superior photocatalytic performance in comparison to TK_400_. Moreover, due to the low carrier mobility of TiO_2_, it is challenging to efficiently drive the photogenerated carriers in the bulk phase of TiO_2_ to the surface to participate in the reaction with a small applied external voltage. The performance of the electric field‐enhanced photocatalytic system was enhanced by a factor of 4 in comparison with un‐hydrogenated TiO_2_.

The stability of the TK_450_ catalyst was evaluated by performing consecutive 10‐cycle catalytic reaction tests under electric field‐enhanced conditions, with each cycle lasting 3 h for a total duration of 30 h. As shown in Figure 4c, the catalytic activity of the TK_450_ catalyst exhibited no significant decay over 10 cycles, maintaining a high level throughout, which demonstrates its stable activity retention capability during repeated use. Furthermore, long‐term continuous operation tests were conducted to assess durability. In the electric field‐enhanced photocatalytic system, the CO_2_ conversion efficiency was monitored during a continuous 10 h operation. As illustrated in Figure 4d, no obvious decline in CO_2_ conversion efficiency was observed over the 10 h period, indicating that the catalyst can maintain excellent catalytic performance under prolonged reaction conditions, further confirming its durability. These results collectively demonstrate that the TK_450_ catalyst exhibits robust stability and exceptional durability in electric field‐enhanced photocatalytic systems, both during repeated cycling and long‐term continuous operation, providing critical evidence for its practical application. Isotopic tracing experiments using ^13^CO_2_ further confirmed that the reduction products originated from ^13^CO_2_. Signals corresponding to ^13^CH_4_ and ^13^C_2_H_6_ were observed at m/z = 17 and 32, respectively (Figure 4e,f), while signals at m/z = 15, 16, 30, and 31 were attributed to ionic fragments of CH_4_ and C_2_H_6_ [37, 38].

### 2.4 Electric Field‐Driven Photogenerated Carrier Separation

**FIGURE 5:**
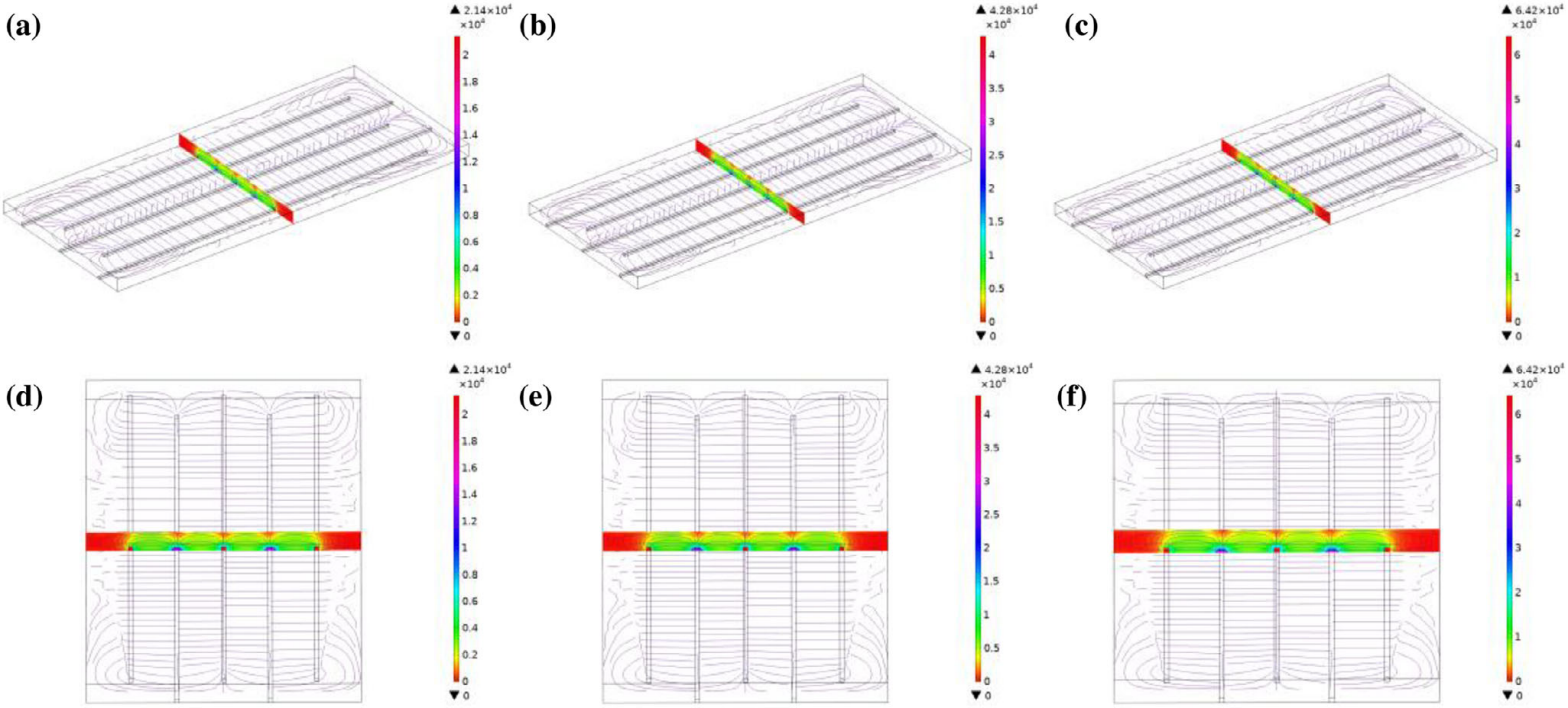
Simulation diagram of electric field intensity.

​Finite element simulations of the electric field distribution within the IDEs under applied biases reveal a pronounced concentration of electric field intensity along the electrode edges, demonstrating a characteristic edge enhancement effect. As depicted in Figure 5, the electric field norm distribution at varying heights above the electrode surface indicates a gradual attenuation of field strength with increasing distance from the electrode. At a given height, the electric field reaches its maximum along the electrode peripheries, while diminishing to a minimum at the center of the inter‐electrode spacing.

Specifically, under a 0.5 V bias (Figure 5a,d), the peak electric field intensity is 1.27 × 10^4^ V m^−1^. When the bias is increased to 1.0 V (Figure 5b,e) and 1.5 V (Figure 5c,f), the maximum field strengths rise to 4.28 × 10 and 6.42 × 10^4^ V m^−1^, respectively. These results clearly indicate that the IDEs under a 1.5 V bias yield the highest electric field strength, confirming a positive correlation between the electric field magnitude and the applied voltage. This behavior aligns well with the fundamental principles of electrostatic theory, which predict a linear field‐voltage response.

**FIGURE 6:**
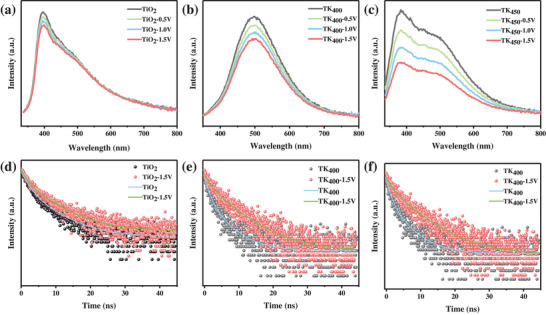
The operando PL spectra of a) pristine TiO_2_; b) TK_400_ and c) TK_450_ under varying applied voltages (0–1.5 V); Operando time‐resolved fluorescence emission decay spectra of d) TiO_2_, e) TK_400_ and f) TK_450_ with the different voltages.

To investigate the impact of external bias voltage on the dynamics of photogenerated carriers, the separation characteristics and lifetimes of charge carriers were systematically analyzed through the operando steady‐state PL and time‐resolved TRPL measurements (Figure S3) [39]. The PL spectra of TiO_2_, TK_400_, and TK_450_ under varying applied voltages (0–1.5 V) were collected to evaluate their carrier recombination behaviors [40]. As depicted in Figure 6a–c, broad visible photoluminescent signals spanning 400–800 nm were observed, attributable to radiative and/or non‐radiative recombination processes mediated by defect‐associated luminescent centers. Quantitative analysis revealed distinct voltage‐dependent PL quenching behaviors. Upon increasing the applied voltage from 0 to 1.5 V, TK_450_ exhibited a significantly more pronounced PL intensity reduction compared to TK_400_ and pristine TiO_2_, which can be attributed to its superior electrical conductivity and enhanced charge carrier separation efficiency. Our previous measurements revealed that hydrogenation‐induced Ti^3+^ doping and oxygen vacancy formation in TK_450_ increased its carrier concentration by 1.9‐fold and carrier mobility by 2.4‐fold relative to undoped TiO_2_ (Table 1). These defects act as fast charge‐transfer channels, reducing bulk recombination rates. Under an external electric field, the directional migration of photogenerated electrons and holes is further accelerated, suppressing radiative recombination pathways. Consequently, TK_450_ demonstrates superior photogenerated carrier utilization efficiency. Under illumination, photogenerated holes are preferentially trapped by bulk OVs introduced via self‐doping, while mobile electrons are attracted to these trapped holes via electrostatic interactions, thereby promoting recombination. In contrast, the application of an external electric field drives electrons to migrate in the opposite direction to holes. This electric field‐induced directional transport, generating a photocurrent and achieving spatial separation of electron‐hole pairs, which effectively mitigates their recombination [41].

**TABLE 1:** Physical parameters of the catalyst.

Sample	N_D_ (cm^−3^)	σ (S cm^−1^)	μ (cm^2^ V^−1^ s^−1^)
TK_450_	4.09 × 10^19^	68.84	10.52
TK_400_	2.73 × 10^19^	31.23	7.15
TiO_2_	2.17 × 10^19^	15.35	4.42

On the other hand, the TRPL spectra of catalysts exhibited analogous characteristics (Figure 6d–f), which were well‐fitted using a biexponential decay model to derive two distinct photoluminescence lifetimes: τ_1_ and τ_2_ in Equation 4 [42, 43, 44]:

(2) $$I\left(t\right)=A_{1}\exp\left(-t/\tau_{1}\right)+A_{2}exp\left(-t/\tau_{2}\right)$$

Herein, τ_1_ corresponds to the radiative recombination lifetime of direct excitonic transitions between bands, whereas τ_2_ originates from the indirect recombination lifetime of trapped carriers at defect states with their oppositely charged counterparts. The associated preexponential factors, A_1_ and A_2_, quantitatively represent the relative contributions of these two recombination pathways [45]. For pristine TiO_2_, the short‐lived component A_1_ dominated with a proportion of 77.1%, indicating that direct excitonic recombination prevails in defect‐free TiO_2_. In contrast, both TK_400_ and TK_450_ exhibited higher proportions of the long‐lived component A_2_ 55.1% for TK_400_ and 57.0% for TK_450_, demonstrating that indirect recombination via defect‐trapped carriers governs their charge dynamics. Notably, upon applying a 1.5 V bias to TiO_2_, TK_400_, and TK_450_, the indirect recombination lifetime τ_2_ increased from 7.6 ns to 8.9 ns and 21.2 ns, respectively, while the corresponding A_2_ proportion rose significantly from 23.3% to 83.5% (Table 2). This voltage‐induced enhancement in τ_2_ and A_2_ highlights the critical role of external electric field in modulating defect‐mediated recombination. Furthermore, self‐doping prolonged the weighted average lifetime (τ) from 5.1 ns in TiO_2_ to 20.7 ns in TK_450_, corroborating that the external electric field effectively delayed the recombination of photo‐generated carriers. The photo‐generated charge separation performance was positively correlated with the carrier mobility. Combined with steady‐state PL analysis, these results strongly suggest that external voltage application in TK_450_ substantially suppresses the recombination of mobile electrons with OV‐trapped holes. Specifically, the applied voltage not only mitigates the trapping of free carriers by self‐doping‐induced defects but also spatially separates electrons and holes via electric field‐driven transport, thereby reducing both free carrier and trap‐assisted recombination.

**TABLE 2:** Parameters from time‐resolved photoluminescence decay curves by fitting a biexponential decay model.

Sample	Voltage (V)	τ_1_ (ns)	A_1_ (%)	τ_2_ (ns)	A_2_ (%)	τ (ns)
TiO_2_	0	1.80	77.14	7.01	22.86	4.59
	1.5	1.72	76.73	7.56	23.27	5.06
TK_400_	0	2.03	44.88	7.61	55.12	6.61
	1.5	1.63	25.08	8.93	74.93	8.51
TK_450_	0	2.07	43.04	7.94	56.96	6.97
	1.5	3.08	16.48	21.17	83.52	20.67

**FIGURE 7:**
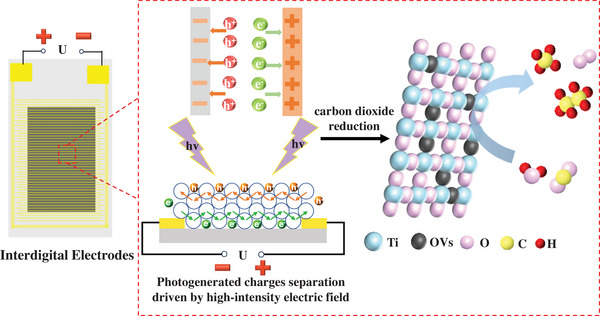
Schematic of photo‐generated carrier migration.

Due to the predominant delocalization of photogenerated electrons within the bulk catalyst and their rapid recombination at room temperature [46], the application of an external voltage is essential to extract these carriers and generate a substantial photocurrent, thereby enhancing photocatalytic performance. Under the irradiation, photogenerated holes are trapped by bulk OVs, forming strong electrostatic interactions with free electrons and driving their migration toward the holes, which exacerbates carrier recombination. Under modest applied voltages (0.5–1.5 V), micro‐spaced IDEs generate high‐intensity electric fields (>10^4^ V/m) through precisely controlled 100 µm gap structures. In such fields, elevated carrier mobility extends the drift length of photogenerated electrons and holes, promoting their migration to catalytic surfaces (Figure 7). In addition, the carrier density determines the initial photo‐generated carrier yield, enabling more photo‐generated carriers to migrate to the catalytic interface and participate in the reaction under the same light intensity. Critically, external electric field enable free electrons to overcome electrostatic attractions and migrate opposite to holes under field‐driven forces, achieving spatial charge separation and suppressing recombination [47]. When applied to flexible IDEs coated with catalyst films, a parallel circuit configuration ensures uniform voltage distribution across the electrode‐catalyst system. Photocarriers conduct along the catalyst/substrate interface, enabling efficient charge transport. For a comprehensive and objective performance evaluation, the catalytic data of various state‐of‐the‐art externally enhanced strategies (e.g., built‐in electric field, applied bias, photothermal catalysis) are compiled in Table S2 for comparison. The benchmarking results highlight that our TK_450_ catalyst achieves a remarkable enhancement of hydrocarbons production under external electric field, outperforming most of the reported counterparts and underscoring its great potential in photo‐electrocatalytic CO_2_ reduction reaction.

## 3 Conclusion

In summary, this study constructs an electric field‐enhanced photocatalytic system for gas‐phase CO_2_ reduction, integrating defective TiO_2_ catalysts with micro‐spaced IDEs. Applied voltages of 0.5–1.5 V across IDEs gaps of 100 µm generate high‐intensity electric fields to achieve the order of 10^4^ V m^−1^ at 1.0 V, which drive directional migration of photogenerated charge carriers. The results demonstrate that photogenerated carrier recombination is significantly suppressed through external electric field application, with photo‐excited electron–hole pairs being expelled from the catalyst to achieve spatial separation of charge carriers. Carrier concentration and mobility in TiO_2_ were enhanced through hydrogenation, achieved by introducing Ti^3+^ species and oxygen vacancies to optimize the semiconductor's electronic structure and facilitate charge carrier transport. Hall effect and Mott–Schottky measurements revealed a 2‐fold increase in charge carrier mobility and a 4.5‐fold enhancement in conductivity for the TK_450_, compared to pristine TiO_2_. Application of an external bias voltage (0.5–1.5 V) induced directional charge migration, suppressing recombination via electric field modulation. This approach enhanced process efficiency fourfold compared to standalone photocatalysis while maintaining product selectivity. The optimized system achieved a CH_4_ production rate of 31.1 µmol g^−1^ h^−1^ and a C_2_H_6_ production rate of 4.9 µmol g^−1^ h^−1^ with exceptional stability over 10 consecutive cycles (30 h). This integrated electrode design eliminates the requirement for conventional photoelectrochemical cells while enabling gas‐phase operation. The noncontact electric field control establishes a practical framework for efficient gas‐phase photocatalytic systems.

## 4 Experimental Section

Refer to the Supporting Information for the reagents, materials, and the general characterization.

### 4.1 Preparation of Photocatalytic Films

The TK_400_ and TK_450_ catalysts were synthesized as follows: 2.5 g of NaBH_4_, 98% and 2.5 g of TiO_2_ were homogenized by grinding in an agate mortar for 5 min. The mixed powder was loaded into a corundum boats and subjected to pyrolysis in a tube furnace under a nitrogen flow (0.1 L min^−1^). The thermal profile comprised a heating ramp from 25°C to the target temperature 400°C or 450°C at 5°C min^−1^, followed by a 30 min isothermal hold. The samples were washed three times with ultrapure water later vacuum‐dried at 60°C for 12 h. The prepared catalysts were denoted as TK_X_ (where “TK” represents the TiO_2_/NaBH_4_ mass ratio of 1:1, and “X” indicates the pyrolysis temperature in °C).

0.1 g of the synthesized catalyst (TK_400_ or TK_450_) was dispersed in a mixed solvent containing 4 mL deionized water and 1 mL absolute ethanol. The resulting homogeneous slurry was then drop‐casted onto 25‐pair interdigitated electrodes (PI substrate, 10 mm × 20 mm, 100 µm line width/spacing) and dried under an infrared lamp to form a stable photocatalytic film.

### 4.2 Photoelectrocatalytic CO_2_ Reduction

Photocatalytic CO_2_ reduction experiments were conducted in a self‐designed 10 mL PEEK reactor equipped with an IDEs coated with the catalyst (Figure S1). The reaction was carried out under irradiation from a UV‐LED light source (365 ± 10 nm), positioned 5 cm away from the reactor. Prior to each experiment, moist high‐purity CO_2_ was introduced into the reactor at a flow rate of 50 mL min^−1^ for 5 min to ensure a CO_2_‐saturated environment. For the photocatalytic CO_2_ reduction experiments under applied external electric fields, wires were connected to the positive and negative terminals of the interdigitated electrodes through pre‐drilled access ports. A regulated DC power supply was used to apply voltages ranging from 0.5 to 1.5 V. Quantitative analysis of gaseous products was performed using a gas chromatograph (GC2014, Shimadzu, Japan). The detailed parameters were provided in the Supporting Information. The apparent quantum yield (AQY) was calculated using the following formula [23]:

(3) $$AQY\left(\%\right)=\frac{N\left(Hydrocarbon\right)\times n\times N_{A}}{N_{p}}\times 100\%$$

(4) $$N_{p}\equiv \frac{H\times A\times \lambda }{h\times c}\times t$$

where N(Hydrocarbon) and n represent the number of moles of hydrocarbon produced from CO_2_ reduction and the number of electrons required, respectively, N_A_ is the Avogadro constant (6.022 × 10^23^ mol^−1^), N_p_ is the number of photons incident, H is the average irradiation intensity (60 mW cm^−2^), A is the irradiated area (0.7 cm^2^), h is the Planck constant (6.626 × 10^−34^ J s), c is the speed of light (3 × 10^8^ m s^−1^), and t is the reaction time.

To analyze the electrostatic characteristics of interdigitated electrodes, a 3D simulation model was established based on the COMSOL Multiphysics 6.3 software platform. The detailed modeling and solving procedures were as follows: First, a geometric model was constructed, comprising the interdigitated electrode structure and the substrate. The electrode material was selected as copper, with an electrical conductivity set to 5.96 × 10^7^ S m^−1^. The substrate material was polyethylene terephthalate (PET), with a relative permittivity of 3.3 and an electrical conductivity of 1 × 10^−14^ S m^−1^, representing its insulating state. For the physics and boundary condition settings, the Electrostatics (ES) interface within the “Electric Fields and Currents” module was employed to simulate the electric field behavior. Displacement currents and Joule heating effects were neglected under the steady‐state electrostatics assumption. The boundary conditions were configured as follows: the entire left set of interdigitated electrodes was defined as a “Terminal” boundary with a applied potential of 0 V; the entire right set was similarly defined as a “Terminal” boundary, with applied potentials of 0.5, 1.0, and 1.5 V, respectively, to simulate different operating conditions; all external surfaces of the substrate and noncontact regions with the electrodes were set as “Zero Charge” boundaries. Subsequently, meshing was performed using free tetrahedral elements to discretize the computational domain. A differentiated mesh control strategy was applied to different components to improve computational efficiency while maintaining accuracy. Finally, a stationary study was selected to solve the governing electrostatic equation (Poisson's equation), and a direct solver (PARDISO) was used to ensure convergence and computational stability. Upon completion of the simulation, the electric field strength distribution cloud plots and vector plots (in V/m) were exported. The maximum electric field strength values on the electrode surface were extracted to provide a basis for insulation design and electric field optimization.

## Author Contributions

X.T. contributed to data curation, investigation, writing – original draft, and formal analysis; Z.Y. contributed to validation and methodology; J.L. contributed to investigation; X.W. and Q.S. contributed to writing – review; H.P. contributed to funding acquisition, project administration, and writing – review and editing; X.L. contributed to resources and writing – review; and P.T. contributed to visualization and writing – review and editing.

## Conflicts of Interest

The authors declare no conflict of interest.

## Supporting information




**Supporting File**: advs73610‐sup‐0001‐SuppMat.docx.

## Data Availability

The data that support the findings of this study are available from the corresponding author upon reasonable request.
