# Supplementary material for: Enhanced Photocatalytic CO2 Reduction Performance by External Electric Field‐Driven Charge Separation on Interdigitated Micro‐Spacing Structure
Source: Adv Sci (Weinh). 2026 Jan 11;13(14):e23076. doi: 10.1002/advs.202523076 (PMC12970266; doi:10.1002/advs.202523076)
Supplement: Supplementary file 1 — Supporting File: advs73610‐sup‐0001‐SuppMat.docx. [file ADVS-13-e23076-s001.docx]

**Supporting Information**

**Enhanced photocatalytic CO_2_ reduction performance by external electric field-driven charge separation on interdigitated micro-spacing structure**

*Xidan Tang^a^, Zhenyu Yu^a^, Jie Liang^a^, Xiaoguang Wang^c^, Qin Shi^a^, Honghui Pan^*,a^, Xixiang Liu^*,a^, Pengyi Tang^*,b^*

**Materials**

All chemicals were used as received without further purification: Titanium dioxide (TiO_2_, P25, 99.8 %), and anhydrous ethanol (C_2_H_5_OH, ≥99.7 %) were purchased from Shanghai Macklin Biochemical Co., Ltd. A 5 wt% Nafion membrane solution was procured from Suzhou Zhengtianruo Scientific New Materials Co., Ltd. Sodium borohydride (NaBH_4_, 98 % purity) was obtained from Chengdu Jingshan Chemical Reagent Co., Ltd. Deionized water (resistivity: 18.25 MΩ·cm) was prepared using a Heal Force NW ultrapure water purification system. The interdigitated flexible electrodes (10 mm × 20 mm, 20 pairs with 100 μm line width and spacing) were purchased from Prisys Biotechnology Co., Ltd.

**Characterization**

The phase composition and crystal structure of the catalysts were characterized using X-ray diffraction (XRD, Bruker D8 Advance, Germany). High-resolution transmission electron microscopy (HR-TEM, JEOL JEM-2100F, Japan) was employed to investigate the morphology and microstructure of the catalysts at an accelerating voltage of 300 kV. Surface chemical composition and valence states were analyzed using X-ray photoelectron spectroscopy (XPS, Thermo Fisher Scientific K-Alpha, USA). The optical properties of the samples were evaluated by ultraviolet-visible diffuse reflectance spectroscopy (UV-vis DRS, SolidSpec-3700), with absorption spectra recorded in the range of 200–1000 nm. Time-resolved photoluminescence (TRPL) spectra were acquired using a fluorescence spectrometer (Edinburgh Instruments, UK) with a 325 nm pulsed laser as the excitation source, and emission signals were collected at 525 nm. For XPS analysis, all spectra were calibrated using the C 1s peak at 284.8 eV as a reference to ensure accurate binding energy measurements

Electrochemical impedance spectroscopy (EIS) was performed using an electrochemical workstation under an AC voltage of 0.005 V with a frequency range of 0.1 MHz to 0.1 Hz. The measurements were conducted to analyze the charge transfer and interfacial properties of the catalysts. According to Mott-Schottky theory, the space charge layer capacitance (as shown in Eq. (1)​) of a semiconductor under depletion conditions follows the relationship^[1]^:

$\frac{1}{Csc^{2}}=\frac{2}{\varepsilon\varepsilon_{0}qN_{D}}\left( U-U_{fb}-\frac{k_{B}T}{q} \right)$ (1)

Where ε is the relative permittivity (for TiO_2_, ε=10), ε_0_ is the vacuum permittivity (8.85×10^−12^  Fm^−1^),q is the elementary charge (1.60×10^−19^  C), N_D_ is the carrier concentration, U is the applied potential, $U_{fb}$ is the flat-band potential, k_B_​ is the Boltzmann constant (1.38×10^−23^ J K^−1^), and T is the temperature in Kelvin (298 K). The carrier concentration (N_D_) was determined by fitting the linear region of the Mott-Schottky plot corresponding to the depletion layer.

Electrochemical tests were carried out in a three-electrode system, consisting of the working electrode (the sample under investigation), a platinum counter electrode, and a silver/silver chloride (Ag/AgCl) reference electrode. A 0.1 M Na_2_SO_4_ aqueous solution was used as the electrolyte. The potential was scanned from -0.4 V to 0.4 V (vs. Ag/AgCl) at a frequency of 1500 Hz. The temperature was maintained at 25 °C using a thermostatically controlled water bath.

The catalyst slurry was prepared by dissolving 20 mg of polyvinyl alcohol (PVA) in a solvent mixture containing 0.8 mL deionized water and 0.2 mL absolute ethanol under continuous magnetic stirring at 80 °C for 30 min. Subsequently, 20 mg of the synthesized catalyst (TK_400_ or TK_450_) was uniformly dispersed into the PVA solution via ultrasonication. The resulting homogeneous slurry was then drop-casted onto 20-pair interdigitated flexible electrodes (PI substrate, 10 mm × 20 mm, 100 μm line width/spacing) and dried under an infrared lamp to form a stable catalytic layer.

The gaseous products generated during the reaction were separated using an SH-Alumina BOND/Na_2_SO_4_ chromatographic column (30 m length, 0.53 mm internal diameter, 10 μm film thickness). Quantitative analysis was performed using a gas chromatograph (GC) equipped with a thermal conductivity detector (TCD) and a flame ionization detector (FID). The TCD was used for the quantification of H_2_ and O_2_ where its temperature was fixed at 200 °C, the oven temperature was maintained at 45 °C and the flow rate of the carrier gas was 5.0 ml min^−1^. The FID was used for the analysis of hydrocarbons where its temperature was fixed at 200 °C, the oven temperature was maintained at 80 °C and the flow rate of the carrier gas was 10 ml min^−1^.

^13^CO_2_ isotope labeling experiments were conducted to track the reaction products. The generated ^13^CH_4_ and ^13^C_2_H_6_ were analyzed using gas chromatography–mass spectrometry (GC–MS) equipped with an HP-PLOT/Q column. The oven temperature was maintained at 70 °C, and high-purity helium was employed as the carrier gas at a flow rate of 10 mL·min^-1^. Prior to the reaction, the entire reaction chamber was purged with humidified ^13^CO_2_ gas.​​

The operando time-resolved photoluminescence (TRPL, FS1000, Edinburgh Instruments, UK) measurements were conducted at excitation wavelengths of 325 nm, with emission monitored at 380 nm (for TK_450_), 400 nm (for TiO_2_) and 500 nm (for TK_400_), respectively. The operando steady-state photoluminescence (PL) spectra were acquired using a spectrofluorometer equipped with a 325 nm excitation source. Prior to each measurement, copper wires were connected to conductive points at both ends of the interdigital electrode. External voltage bias was applied and regulated via a steady-state DC power supply.

**Figure S1.** Schematic diagram of the experimental setup used for electric field-enhanced photocatalytic CO_2_ reduction.

**Figure S2.** TEM images of (a) TiO_2_, (b) TK_400_ and (c) TK_450_.


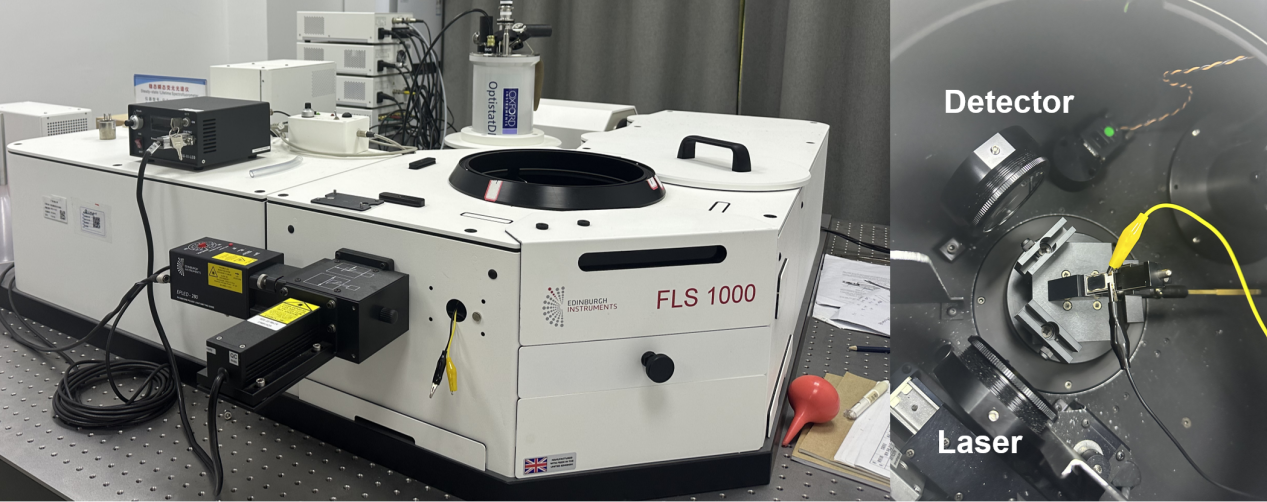


**Figure S3.** Depiction of monitoring device used for the operando steady-state PL measurement and TRPL spectra measurement.

**Figure S4.** Control test results of sample TK_450_.

**Figure S5.** XPS survey spectra of all prepared catalysts.

**Figure S6.** Rate of hydrocarbons production for each catalyst applied by different external voltages.

**Table S1** relative peak area (P.A.) of Ti 2p, and molar percentages (M.P.) of Ti^3+^ and Ti^4+^ species of the catalysts.

| Catalyst | B.E(eV） | | | | Peak Area（Counts·eV） | | | | M.P(%) | |
| --- | --- | --- | --- | --- | --- | --- | --- | --- | --- | --- |
|  | Ti^4+^ | | Ti^3+^ | | Ti^4+^ | | Ti^3+^ | | Ti^4+^ | Ti^3+^ |
|  | 2p_1/2_ | 2p_3/2_ | 2p_1/2_ | 2p_3/2_ | 2p_1/2_ | 2p_3/2_ | 2p_1/2_ | 2p_3/2_ |  |  |
| TK_450_ | 464.6 | 458.7 | 463.8 | 458.2 | 487.1 | 156.0 | 659.6 | 1917.1 | 20.0 | 80.0 |
| TK_400_ | 464.3 | 458.3 | 463.6 | 457.9 | 615.4 | 441.0 | 768.9 | 1522.2 | 31.6 | 68.4 |
| TiO_2_ | 464.5 | 458.5 | 463.7 | 458.1 | 808.2 | 465.3 | 539.3 | 1218.5 | 42.0 | 58.0 |

**Table S2** Recent reports on studies for enhancing photocatalytic performance via external fields

| Catalysts | External Field | Rate of production | References |
| --- | --- | --- | --- |
| Rutile | Alternating current electric field | CH_4_:2.1 μmol·g^-1^ h^-1^ | ^[2]^ |
| 5NaH_2_PO_4_-20TiO_2_/g-C_3_N_4_ | Built-in electric field | AQY:0.288 % | ^[3]^ |
| CoOx-TiO_2_ | ​External DC electric field​​ | CH₃OH:56.6 μmol cm^-2^ h^-1^ | ^[4]^ |
| RuO_2_(6.4%):TiO_2_(16.9%)/SBA-15 | Thermo-photocatalysis | CH_4_:58.6 mmol g^-1^_active cat._ h^-1^ | ^[5]^ |
| CuO/TNTs | Applied potential | CH₃OH:30 μmol cm^-2^ | ^[6]^ |
| TK_450_ | ​External Electric Field​​ | CH_4_:31.1 μmol·g^-1^ h^-1^ | This·work. |

**References**

[1] H. Zhou, Y. Zhang, *J. Phys. Chem. C.* **2014**, *118*, 5626.

[2] O. Shtyka, R. Ciesielski, A. Kedziora, M. Szynkowska-Jozwik, T. Maniecki, *Catal Lett.* **2025**, *155*, 46.

[3] M. Chu, Y. Li, K. Cui, J. Jian, S. Lu, P. Gao, X. Wu, *Environ Chem Lett.* **2022**, *20*, 999.

[4] D. Pan, X. Ye, Y. Cao, S. Zhu, X. Chen, M. Chen, D. Zhang, G. Li, *Applied Surface Science.* **2020**, *511*, 145497.

[5] L. O. Paulista, A. F. P. Ferreira, B. Castanheira, M. B. Ðolić, R. J. E. Martins, R. A. R. Boaventura, V. J. P. Vilar, T. F. C. V. Silva, *​​Appl. Catal. B :Environ.​​* **2024**, *340*, 123232.

[6] L. Zhang, H. Cao, Q. Pen, L. Wu, G. Hou, Y. Tang, G. Zheng, *Electrochimica Acta.* **2018**, *283*, 1507.
